# 5-Fluoro-2-(4-iodo­phen­yl)-3-methyl­sulfinyl-1-benzofuran

**DOI:** 10.1107/S1600536809030992

**Published:** 2009-08-08

**Authors:** Hong Dae Choi, Pil Ja Seo, Byeng Wha Son, Uk Lee

**Affiliations:** a Department of Chemistry, Dongeui University, San 24 Kaya-dong Busanjin-gu, Busan 614-714, Republic of Korea; b Department of Chemistry, Pukyong National University, 599-1 Daeyeon 3-dong, Nam-gu, Busan 608-737, Republic of Korea

## Abstract

In the title compound, C_15_H_10_FIO_2_S, the O atom and the methyl group of the methyl­sulfinyl substituent lie on opposite sides of the plane through the benzofuran fragment. The 4-iodo­phenyl ring is rotated out of the benzofuran plane by a dihedral angle of 39.4 (1)°. The crystal structure is stabilized by an inter­molecular C—H⋯O hydrogen bond and an I⋯O halogen bond [3.055 (2) Å]. The crystal structure also exhibits an inter­molecular C—H⋯π inter­action between the methyl H atom and the 4-iodo­phenyl ring of an adjacent benzofuran mol­ecule, and aromatic π–π inter­actions between the benzene rings of neighbouring benzofuran systems [centroid–centroid distance = 3.558 (3) Å].

## Related literature

For the crystal structures of similar 2-(4-iodo­phen­yl)-3-methyl­sulfinyl-1-benzofuran derivatives, see: Choi *et al.* (2008*a* ▶,*b* ▶). For the pharmacological activity of benzofuran compounds, see: Howlett *et al.* (1999 ▶); Twyman & Allsop (1999 ▶). For a review of halogen bonding, see: Politzer *et al.* (2007 ▶).
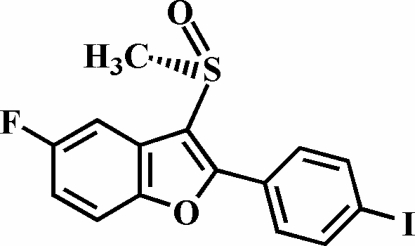


## Experimental

### 

#### Crystal data

C_15_H_10_FIO_2_S
*M* *_r_* = 400.19
Triclinic, 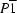

*a* = 8.8989 (5) Å
*b* = 9.2370 (5) Å
*c* = 10.3357 (5) Å
α = 105.579 (1)°
β = 115.302 (1)°
γ = 101.671 (1)°
*V* = 689.08 (6) Å^3^
*Z* = 2
Mo *K*α radiation
μ = 2.48 mm^−1^
*T* = 273 K
0.25 × 0.15 × 0.10 mm

#### Data collection

Bruker SMART CCD diffractometer
Absorption correction: multi-scan (*SADABS*; Sheldrick, 2000 ▶)) *T*_min_ = 0.650, *T*_max_ = 0.784
5972 measured reflections
2957 independent reflections
2689 reflections with *I* > 2σ(*I*)
*R*_int_ = 0.017

#### Refinement

*R*[*F* ^2^ > 2σ(*F* ^2^)] = 0.022
*wR*(*F* ^2^) = 0.055
*S* = 1.09
2957 reflections
182 parameters
H-atom parameters constrained
Δρ_max_ = 0.68 e Å^−3^
Δρ_min_ = −0.55 e Å^−3^

### 

Data collection: *SMART* (Bruker, 2001 ▶); cell refinement: *SAINT* (Bruker, 2001 ▶); data reduction: *SAINT*; program(s) used to solve structure: *SHELXS97* (Sheldrick, 2008 ▶); program(s) used to refine structure: *SHELXL97* (Sheldrick, 2008 ▶); molecular graphics: *ORTEP-3* (Farrugia, 1997 ▶) and *DIAMOND* (Brandenburg, 1998 ▶); software used to prepare material for publication: *SHELXL97*.

## Supplementary Material

Crystal structure: contains datablocks global, I. DOI: 10.1107/S1600536809030992/vm2001sup1.cif

Structure factors: contains datablocks I. DOI: 10.1107/S1600536809030992/vm2001Isup2.hkl

Additional supplementary materials:  crystallographic information; 3D view; checkCIF report

## Figures and Tables

**Table 1:** Hydrogen-bond geometry (Å, °)

*D*—H⋯*A*	*D*—H	H⋯*A*	*D*⋯*A*	*D*—H⋯*A*
C15—H15*C*⋯O2^i^	0.96	2.42	3.238 (3)	143
C15—H15*B*⋯*Cg*3^ii^	0.96	2.91	3.554 (3)	126

Symmetry codes: (i) 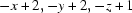; (ii) 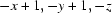. *Cg*3 is the centroid of the C9–C14 benzene ring.

**Fig. 1.**
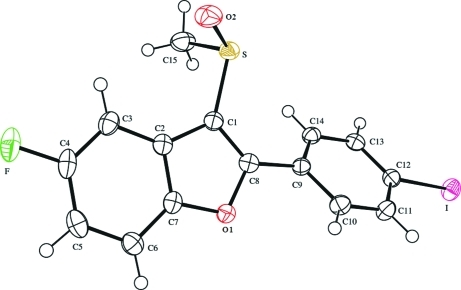
The molecular structure of the title compound with the atom numbering scheme. Displacement ellipsoids are drawn at the 50% probability level. H atoms are presented as a small spheres of arbitrary radius.

**Fig. 2.**
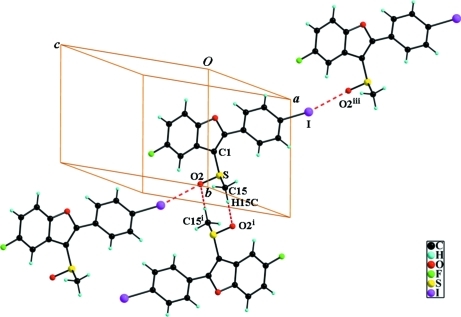
C–H···O and C–I···O interactions (dotted lines) in the crystal structure of title compound. [Symmetry code: (i) - x + 2, - y + 2, - z + 1; (iii) x, y - 1, z - 1.]

**Fig. 3.**
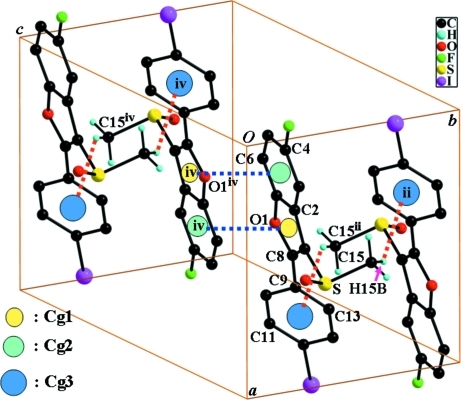
C–H···π and π–π interactions (dotted lines) in the crystal structure of title compound. Cg denotes the ring centroids. [Symmetry code: (ii) - x + 1, - y + 1, - z; (iv) - x + 1, - y + 1, - z + 1.]

## supplementary crystallographic information

### Comment

The benzofuran ring systems have received considerable attention in the field of their biological and pharmacological properties (Howlett *et a*l., 1999; Twyman & Allsop, 1999). This work is related to our communications on the synthesis and structures of 2-(4-iodophenyl)-3-methylsulfinyl-1-benzofuran analogues, *viz*. 2-(4-iodophenyl)-5-methyl-3-methylsulfinyl-1-benzofuran (Choi *et al.*, 2008a) and 2-(4-iodophenyl)-5,7-dimethyl-3-methylsulfinyl-1-benzofuran (Choi *et al.*, 2008*b*). Here we report the crystal structure of the title compound (Fig. 1).

The benzofuran unit is essentially planar, with a mean deviation of 0.010 (2) Å from the least-squares plane defined by the nine constituent atoms. The dihedral angle formed by the planes of the benzofuran and the 4-iodophenyl rings is 39.4 (1)°. The crystal packing (Fig. 2) is stabilized by an intermolecular C–H···O hydrogen bond and an I···O halogen bond (Politzer *et al.*, 2007); the first between the methyl H atom and the S═O unit, with a C15–H15C···O2^i^ distance of 3.238 (3) Å (Table 1), the second between the iodine atom and the oxygen of the S═O unit, *i.e*. an I···O distance of 3.055 (2) Å and a nearly linear C12–I···O2^iii^ angle of 165.26 (8)°. The crystal packing (Fig. 3) also exhibits an intermolecular C–H···π interaction between the methyl H atom and the 4-bromophenyl ring of an adjacent molecule, with a C15–H15B···Cg3^ii^ (Table 1; Cg3 is the centroid of the C9-C14 benzene ring). The further stability comes from aromatic π–π interaction between the furan and the benzene rings of the adjacent molecules, with a Cg1···Cg2^iv^ distance of 3.558 (3) Å (Fig. 3; Cg1 and Cg2 are the centroids of the C1/C2/C7/O2/C8 furan ring and the C2-C7 benzene ring, respectively).

### Experimental

The 77% 3-chloroperoxybenzoic acid (291 mg, 1.3 mmol) was added in small portions to a stirred solution of 5-fluoro-2-(4-iodophenyl)-3-methylsulfanyl-1-benzofuran (310 mg, 1.2 mmol) in dichloromethane (30 mL) at 273 K. After being stirred at room temperature for 3h, the mixture was washed with saturated sodium bicarbonate solution and the organic layer was separated, dried over magnesium sulfate, filtered and concentrated in vacuum. The residue was purified by column chromatography (hexan-ethyl acetate, 1 : 2 v/v) to afford the title compound as a colorless solid [yield 81%, m.p. 482-483 K; *R*_f_ = 0.71 (hexane-ethyl acetate, 1:2 v/v)]. Single crystals suitable for X-ray diffraction were prepared by slow evaporation of a solution of the title compound in tetrahydrofuran at room temperature.

### Refinement

All H atoms were positioned geometrically and refined using a riding model, with C–H = 0.93 Å for aromatic H atoms and 0.96 Å for methyl H atoms, respectively, and with *U*_iso_(H) = 1.2*U*_eq_(C) for aromatic H atoms and 1.5 *U*_eq_(C) for methyl H atoms, respectively.

### Crystal data

**Table:**

C_15_H_10_FIO_2_S	*Z* = 2
*M**_r_* = 400.19	*F*(000) = 388
Triclinic, *P*1	*D*_x_ = 1.929 Mg m^−^^3^
Hall symbol: -P 1	Mo *K*α radiation, λ = 0.71073 Å
*a* = 8.8989 (5) Å	Cell parameters from 4508 reflections
*b* = 9.2370 (5) Å	θ = 2.4–27.4°
*c* = 10.3357 (5) Å	µ = 2.48 mm^−^^1^
α = 105.579 (1)°	*T* = 273 K
β = 115.302 (1)°	Block, colorless
γ = 101.671 (1)°	0.25 × 0.15 × 0.10 mm
*V* = 689.08 (6) Å^3^	

### Data collection

**Table:**

Bruker SMART CCD diffractometer	2957 independent reflections
Radiation source: fine-focus sealed tube	2689 reflections with *I* > 2σ(*I*)
graphite	*R*_int_ = 0.017
Detector resolution: 10.0 pixels mm^-1^	θ_max_ = 27.0°, θ_min_ = 2.4°
φ and ω scans	*h* = −11→11
Absorption correction: multi-scan (*SADABS*; Sheldrick, 2000))	*k* = −11→11
*T*_min_ = 0.650, *T*_max_ = 0.784	*l* = −13→13
5972 measured reflections	

### Refinement

**Table:**

Refinement on *F*^2^	Primary atom site location: structure-invariant direct methods
Least-squares matrix: full	Secondary atom site location: difference Fourier map
*R*[*F*^2^ > 2σ(*F*^2^)] = 0.022	Hydrogen site location: difference Fourier map
*wR*(*F*^2^) = 0.055	H-atom parameters constrained
*S* = 1.09	*w* = 1/[σ^2^(*F*_o_^2^) + (0.0248*P*)^2^ + 0.3594*P*] where *P* = (*F*_o_^2^ + 2*F*_c_^2^)/3
2957 reflections	(Δ/σ)_max_ = 0.001
182 parameters	Δρ_max_ = 0.68 e Å^−^^3^
0 restraints	Δρ_min_ = −0.55 e Å^−^^3^

### Special details

**Table:**

Geometry. All esds (except the esd in the dihedral angle between two l.s. planes) are estimated using the full covariance matrix. The cell esds are taken into account individually in the estimation of esds in distances, angles and torsion angles; correlations between esds in cell parameters are only used when they are defined by crystal symmetry. An approximate (isotropic) treatment of cell esds is used for estimating esds involving l.s. planes.
Refinement. Refinement of F^2^ against ALL reflections. The weighted R-factor wR and goodness of fit S are based on F^2^, conventional R-factors R are based on F, with F set to zero for negative F^2^. The threshold expression of F^2^ > 2sigma(F^2^) is used only for calculating R-factors(gt) etc. and is not relevant to the choice of reflections for refinement. R-factors based on F^2^ are statistically about twice as large as those based on F, and R- factors based on ALL data will be even larger.

### Fractional atomic coordinates and isotropic or equivalent isotropic displacement parameters (Å^2^)

**Table:**

	*x*	*y*	*z*	*U*_iso_*/*U*_eq_	
I	0.87629 (2)	0.09755 (2)	−0.19147 (2)	0.03012 (7)	
S	0.81950 (8)	0.77048 (7)	0.37848 (7)	0.02442 (13)	
F	0.2583 (2)	0.7621 (2)	0.5294 (2)	0.0428 (4)	
O1	0.4384 (2)	0.3431 (2)	0.1980 (2)	0.0233 (4)	
O2	0.8909 (3)	0.8594 (2)	0.5482 (2)	0.0356 (4)	
C1	0.6269 (3)	0.6020 (3)	0.3101 (3)	0.0219 (5)	
C2	0.4906 (3)	0.5987 (3)	0.3510 (3)	0.0222 (5)	
C3	0.4518 (3)	0.7126 (3)	0.4387 (3)	0.0264 (5)	
H3	0.5242	0.8214	0.4904	0.032*	
C4	0.3009 (4)	0.6553 (3)	0.4443 (3)	0.0292 (6)	
C5	0.1882 (4)	0.4941 (4)	0.3707 (3)	0.0310 (6)	
H5	0.0871	0.4634	0.3783	0.037*	
C6	0.2275 (3)	0.3798 (3)	0.2861 (3)	0.0269 (5)	
H6	0.1562	0.2709	0.2367	0.032*	
C7	0.3788 (3)	0.4369 (3)	0.2791 (3)	0.0226 (5)	
C8	0.5907 (3)	0.4480 (3)	0.2194 (3)	0.0220 (5)	
C9	0.6706 (3)	0.3730 (3)	0.1356 (3)	0.0215 (5)	
C10	0.6734 (3)	0.2185 (3)	0.1215 (3)	0.0254 (5)	
H10	0.6323	0.1671	0.1724	0.030*	
C11	0.7365 (3)	0.1415 (3)	0.0331 (3)	0.0262 (5)	
H11	0.7377	0.0389	0.0242	0.031*	
C12	0.7985 (3)	0.2194 (3)	−0.0429 (3)	0.0225 (5)	
C13	0.7989 (3)	0.3733 (3)	−0.0283 (3)	0.0225 (5)	
H13	0.8410	0.4247	−0.0786	0.027*	
C14	0.7364 (3)	0.4501 (3)	0.0616 (3)	0.0225 (5)	
H14	0.7384	0.5539	0.0725	0.027*	
C15	0.7072 (4)	0.8817 (3)	0.2787 (3)	0.0356 (6)	
H15A	0.6214	0.8998	0.3076	0.053*	
H15B	0.6475	0.8212	0.1679	0.053*	
H15C	0.7929	0.9835	0.3071	0.053*	

### Atomic displacement parameters (Å^2^)

**Table:**

	*U*^11^	*U*^22^	*U*^33^	*U*^12^	*U*^13^	*U*^23^
I	0.03872 (11)	0.03141 (10)	0.02933 (10)	0.01738 (8)	0.02328 (8)	0.01132 (8)
S	0.0221 (3)	0.0233 (3)	0.0206 (3)	0.0048 (2)	0.0092 (3)	0.0043 (2)
F	0.0485 (11)	0.0531 (11)	0.0389 (10)	0.0314 (9)	0.0292 (9)	0.0139 (8)
O1	0.0238 (9)	0.0208 (8)	0.0252 (9)	0.0074 (7)	0.0141 (7)	0.0070 (7)
O2	0.0340 (11)	0.0350 (11)	0.0230 (9)	0.0030 (9)	0.0127 (8)	0.0015 (8)
C1	0.0206 (12)	0.0228 (12)	0.0181 (11)	0.0074 (10)	0.0076 (10)	0.0065 (10)
C2	0.0212 (12)	0.0263 (12)	0.0165 (11)	0.0101 (10)	0.0071 (10)	0.0084 (10)
C3	0.0302 (13)	0.0279 (13)	0.0194 (12)	0.0138 (11)	0.0112 (11)	0.0073 (10)
C4	0.0329 (14)	0.0414 (15)	0.0209 (12)	0.0251 (13)	0.0149 (11)	0.0126 (12)
C5	0.0256 (13)	0.0466 (16)	0.0299 (14)	0.0187 (12)	0.0161 (12)	0.0206 (13)
C6	0.0239 (13)	0.0313 (14)	0.0264 (13)	0.0110 (11)	0.0117 (11)	0.0140 (11)
C7	0.0249 (12)	0.0263 (12)	0.0185 (11)	0.0132 (10)	0.0102 (10)	0.0100 (10)
C8	0.0225 (12)	0.0239 (12)	0.0188 (11)	0.0076 (10)	0.0096 (10)	0.0097 (10)
C9	0.0195 (12)	0.0198 (11)	0.0188 (11)	0.0058 (9)	0.0074 (10)	0.0042 (9)
C10	0.0287 (13)	0.0248 (12)	0.0257 (13)	0.0093 (11)	0.0150 (11)	0.0128 (11)
C11	0.0317 (14)	0.0218 (12)	0.0289 (13)	0.0117 (11)	0.0169 (11)	0.0113 (11)
C12	0.0215 (12)	0.0238 (12)	0.0190 (11)	0.0074 (10)	0.0104 (10)	0.0046 (10)
C13	0.0205 (12)	0.0253 (12)	0.0183 (11)	0.0050 (10)	0.0090 (10)	0.0079 (10)
C14	0.0214 (12)	0.0203 (11)	0.0206 (12)	0.0064 (10)	0.0076 (10)	0.0072 (10)
C15	0.0327 (15)	0.0257 (14)	0.0391 (16)	0.0067 (12)	0.0117 (13)	0.0141 (12)

### Geometric parameters (Å, °)

**Table:**

I—C12	2.098 (2)	C6—C7	1.383 (3)
I—O2^i^	3.055 (2)	C6—H6	0.9300
S—O2	1.489 (2)	C8—C9	1.463 (3)
S—C1	1.776 (2)	C9—C14	1.394 (3)
S—C15	1.794 (3)	C9—C10	1.402 (3)
F—C4	1.364 (3)	C10—C11	1.381 (4)
O1—C7	1.382 (3)	C10—H10	0.9300
O1—C8	1.386 (3)	C11—C12	1.398 (3)
C1—C8	1.359 (3)	C11—H11	0.9300
C1—C2	1.443 (3)	C12—C13	1.387 (3)
C2—C7	1.395 (3)	C13—C14	1.387 (3)
C2—C3	1.396 (3)	C13—H13	0.9300
C3—C4	1.373 (4)	C14—H14	0.9300
C3—H3	0.9300	C15—H15A	0.9600
C4—C5	1.391 (4)	C15—H15B	0.9600
C5—C6	1.384 (4)	C15—H15C	0.9600
C5—H5	0.9300		
			
C12—I—O2^i^	165.26 (8)	C1—C8—C9	134.3 (2)
O2—S—C1	106.61 (11)	O1—C8—C9	114.9 (2)
O2—S—C15	107.29 (13)	C14—C9—C10	119.0 (2)
C1—S—C15	97.37 (12)	C14—C9—C8	120.6 (2)
C7—O1—C8	106.13 (18)	C10—C9—C8	120.3 (2)
C8—C1—C2	107.4 (2)	C11—C10—C9	121.0 (2)
C8—C1—S	125.10 (19)	C11—C10—H10	119.5
C2—C1—S	127.23 (18)	C9—C10—H10	119.5
C7—C2—C3	119.1 (2)	C10—C11—C12	119.2 (2)
C7—C2—C1	105.1 (2)	C10—C11—H11	120.4
C3—C2—C1	135.9 (2)	C12—C11—H11	120.4
C4—C3—C2	116.3 (2)	C13—C12—C11	120.5 (2)
C4—C3—H3	121.9	C13—C12—I	119.62 (17)
C2—C3—H3	121.9	C11—C12—I	119.79 (18)
F—C4—C3	118.3 (2)	C14—C13—C12	119.9 (2)
F—C4—C5	117.1 (2)	C14—C13—H13	120.1
C3—C4—C5	124.5 (2)	C12—C13—H13	120.1
C6—C5—C4	119.6 (2)	C13—C14—C9	120.5 (2)
C6—C5—H5	120.2	C13—C14—H14	119.8
C4—C5—H5	120.2	C9—C14—H14	119.8
C7—C6—C5	116.2 (2)	S—C15—H15A	109.5
C7—C6—H6	121.9	S—C15—H15B	109.5
C5—C6—H6	121.9	H15A—C15—H15B	109.5
O1—C7—C6	125.1 (2)	S—C15—H15C	109.5
O1—C7—C2	110.6 (2)	H15A—C15—H15C	109.5
C6—C7—C2	124.3 (2)	H15B—C15—H15C	109.5
C1—C8—O1	110.7 (2)		
			
O2—S—C1—C8	−132.7 (2)	C2—C1—C8—O1	0.0 (3)
C15—S—C1—C8	116.8 (2)	S—C1—C8—O1	174.65 (16)
O2—S—C1—C2	40.9 (2)	C2—C1—C8—C9	176.3 (3)
C15—S—C1—C2	−69.7 (2)	S—C1—C8—C9	−9.1 (4)
C8—C1—C2—C7	0.0 (3)	C7—O1—C8—C1	0.0 (3)
S—C1—C2—C7	−174.51 (18)	C7—O1—C8—C9	−177.1 (2)
C8—C1—C2—C3	−179.9 (3)	C1—C8—C9—C14	−37.3 (4)
S—C1—C2—C3	5.6 (4)	O1—C8—C9—C14	138.8 (2)
C7—C2—C3—C4	−1.4 (3)	C1—C8—C9—C10	146.1 (3)
C1—C2—C3—C4	178.5 (3)	O1—C8—C9—C10	−37.8 (3)
C2—C3—C4—F	179.6 (2)	C14—C9—C10—C11	−1.4 (4)
C2—C3—C4—C5	0.5 (4)	C8—C9—C10—C11	175.2 (2)
F—C4—C5—C6	−178.4 (2)	C9—C10—C11—C12	0.1 (4)
C3—C4—C5—C6	0.7 (4)	C10—C11—C12—C13	0.8 (4)
C4—C5—C6—C7	−1.0 (4)	C10—C11—C12—I	−175.29 (19)
C8—O1—C7—C6	178.8 (2)	O2^i^—I—C12—C13	−117.3 (3)
C8—O1—C7—C2	0.0 (2)	O2^i^—I—C12—C11	58.8 (4)
C5—C6—C7—O1	−178.5 (2)	C11—C12—C13—C14	−0.4 (4)
C5—C6—C7—C2	0.1 (4)	I—C12—C13—C14	175.70 (18)
C3—C2—C7—O1	179.9 (2)	C12—C13—C14—C9	−1.0 (4)
C1—C2—C7—O1	0.0 (3)	C10—C9—C14—C13	1.8 (4)
C3—C2—C7—C6	1.1 (4)	C8—C9—C14—C13	−174.8 (2)
C1—C2—C7—C6	−178.8 (2)		

Symmetry codes: (i) *x*, *y*−1, *z*−1.

### Hydrogen-bond geometry (Å, °)

**Table:**

*D*—H···*A*	*D*—H	H···*A*	*D*···*A*	*D*—H···*A*
C15—H15C···O2^ii^	0.96	2.42	3.238 (3)	143
C15—H15B···Cg3^iii^	0.96	2.91	3.554 (3)	126

Symmetry codes: (ii) −*x*+2, −*y*+2, −*z*+1; (iii) −*x*+1, −*y*+1, −*z*.
